# Using cognitive load theory to evaluate and improve preparatory materials and study time for the flipped classroom

**DOI:** 10.1186/s12909-023-04325-x

**Published:** 2023-05-17

**Authors:** Krisztina Fischer, Amy M. Sullivan, Amy P. Cohen, Randall W. King, Barbara A. Cockrill, Henrike C. Besche

**Affiliations:** 1 Department of Radiology, Brigham and Women’s Hospital, Harvard Medical School, Boston, Massachusetts USA; 2 Department of Medicine, Beth Israel Deaconess Medical Center, Harvard Medical School, Boston, Massachusetts USA; 3 Harvard T.H. Chan School of Public Health, Boston, Massachusetts USA; 4 Department of Cell Biology, Harvard Medical School, Boston, Massachusetts USA; 5 Department of Medicine, Brigham and Women’s Hospital, Harvard Medical School, Boston, Massachusetts USA; 6 Program in Medical Education, Harvard Medical School, Boston, Massachusetts USA

**Keywords:** Instructional design, Flipped classroom, Efficiency, Cognitive load theory, Educational quality improvement

## Abstract

**Background:**

Preclinical medical education is content-dense and time-constrained. Flipped classroom approaches promote durable learning, but challenges with unsatisfactory student preparation and high workload remain. Cognitive load theory defines instructional design as “efficient” if learners can master the presented concepts without cognitive overload. We created a PReparatory Evaluation Process (PREP) to systematically assess and measure improvement in the cognitive-load efficiency of preparatory materials and impact on study time (time-efficiency).

**Methods:**

We conducted this study in a flipped, multidisciplinary course for ~ 170 first year students at Harvard Medical School using a naturalistic post-test design. For each flipped session (n = 97), we assessed cognitive load and preparatory study time by administering a 3-item PREP survey embedded within a short subject-matter quiz students completed before class. Over three years (2017–2019), we evaluated cognitive load- and time- based efficiency to guide iterative revisions of the materials by content experts. The ability of PREP to detect changes to the instructional design (sensitivity) was validated through a manual audit of the materials.

**Results:**

The average survey response rate was ≥ 94%. Content expertise was not required to interpret PREP data. Initially students did not necessarily allocate the most study time to the most difficult content. Over time, the iterative changes in instructional design increased the cognitive load- and time-based efficiency of preparatory materials with large effect sizes (*p* < .01). Furthermore, this increased the overall alignment of cognitive load with study time: students allocated more time to difficult content away from more familiar, less difficult content without increasing workload overall.

**Conclusions:**

Cognitive load and time constraints are important parameters to consider when designing curricula. The PREP process is learner-centered, grounded in educational theory, and works independently of content knowledge. It can provide rich and actionable insights into instructional design of flipped classes not captured by traditional satisfaction-based evaluations.

**Supplementary Information:**

The online version contains supplementary material available at 10.1186/s12909-023-04325-x.

## Introduction

In the last decades, preclinical medical education reform has focused on the transition from traditional lecture-based instruction to various forms of “flipped-classroom” teaching to provide students with more experience applying their knowledge and struggling with clinical problems [1–3]. The flipped classroom method aims to align learning better with human cognition and thus make learning deeper and more durable, a goal that is often referred to as “active learning” [4, 5]. The success of the flipped classroom format requires that students arrive well-prepared to participate in class [6]. Ensuring students have adequate time and effective resources to prepare for class have emerged as common challenges in implementing flipped classroom formats [6, 7].

Medical school faculty are not usually trained in instructional design, and, as content experts, may struggle to accurately assess the cognitive difficulty or time required for novice learners to work through assigned materials. This phenomenon is a normal cognitive bias sometimes called the “expert blind spot” [8]. Providing students with overly comprehensive preparatory materials can convince faculty that the content is well covered, but as a result students may be overwhelmed by too much content leading to inadequate preparation for class [9–13] and thus interfere with active learning.

The iterative cycle of curriculum improvement is routinely performed by faculty and requires significant time and resources. While empirical evidence regarding instructional design for the flipped classroom is emerging [14–16], standardized design frameworks are still lacking [17–19]. Satisfaction-based endofcourse evaluations are widely used in higher education to assess teaching, but they lack in granularity to assess effectiveness at the level of day-to-day instructional design [20–22]. Thus, methods to evaluate and improve preparatory resources for flipped classes to promote student preparation for active learning are needed.

To address this problem, we developed a learner-centered PReparatory Evaluation Process (PREP) grounded in cognitive load theory (CLT). CLT defines instruction as “efficient,” when it provides the learner with sufficient guidance to successfully process novel information without overloading the limited capacity of working memory [23, 24]. The level of guidance required depends on both the learner’s prior expertise and the intrinsic complexity of the topic [25, 26]. The efficiency of the instructional materials has typically been assessed by comparing the performance on the learning task with the intensity of mental effort (“difficulty of the material”) in form of efficiency graphs or metrics [27–29]. This method has been widely used in the field of instructional design to assess the cognitive load efficiency of learning tasks with strong psychometric properties in various contexts [27, 29–34]. Given the time-compressed nature of undergraduate medical education and the challenges observed with managing workload in the flipped setting [7, 10], we expanded the traditional notion of cognitive load-based efficiency to also include prep time.

PREP consist of two steps – first measuring instructional efficiency of prep assignments to identify resources in need of revision, second applying instructional design principles derived from CLT [24, 26] to optimize instructional efficiency. To our knowledge, this is the first study to systematically apply CLT to assess how iterative changes to the instructional design affect the self-reported cognitive load and workload of prep resources in a flipped curriculum.

Specifically, we focused on the following research questions: (1) How can the PREP tool be used to assess the cognitive-load and time-based efficiency of individual preparatory materials? (2) What is the sensitivity of the PREP tool in detecting changes in the instructional design of preparatory materials? (3) What is the overall impact of the PREP process on instructional efficiency of the entire course?

## Methods

### Study design

This study describes a naturalistic post-test study without a control group looking at the cognitive load-and time-based efficiency of students engaging with flipped classroom learning in the basic science component of an undergraduate medical program. The Harvard Medical School (HMS) Program in Medical Education (PME) Educational Scholarship Review Committee deemed this study not human subjects research and exempt from further IRB review. The need for written informed consent was waived by the HMS PME Educational Scholarship Review Committee due to the retrospective nature of the study. We followed the revised standards for quality improvement reporting excellence [35].

### Context

This study was conducted in context of a multidisciplinary, pre-clinical basic science course in the *Pathways* program at Harvard Medical School. The 13.5 week-long course was taken by 170 students each year (~ 135 medical and ~ 35 dental students) as part of a long-standing joint first-year program where students were enrolled without differentiation in the same courses. The course, *Foundations*, interleaved 97 individual flipped-classroom sessions in ten different disciplines: cell biology, anatomy, developmental biology, histology, pathology, cancer biology, genetics, immunology, microbiology and pharmacology. Students attended class Monday, Tuesday, Thursday and Friday mornings each week (8:00 AM – 12:30 PM) while the afternoons were reserved for preparatory study and consolidation. Wednesdays were reserved for clinical training (see Appendix 1 for an exemplary week of the course schedule). Faculty recommend that students distribute their preparatory time across the week (including weekends), so that they prepare for no more than two individual sessions per afternoon.

### Instructional design of preparatory resources

The course faculty applied the following design principles to prep work for all flipped classroom sessions: (1) Limit prep work to ~ 2 h study per ~ 80 min in-class session (or ~ 1.5x of in-class time); (2) Provide students with a short summary of the topic (5–6 sentences), learning objectives, and important keywords; (3) Where applicable, organize content derived from prior lectures into several shorter concept videos (typically in form of narrated slide presentations). Otherwise, study resources may vary, ranging from book chapters to articles or other readings; (4) Conclude each preparatory assignment with a short, open-book, multiple choice quiz to provide students with feedback on their preparation (*readiness-assessment exercise* or RAE). Students received credit for each RAE submitted before each session, if ≥ 50% correct. Cumulatively, all RAEs accounted for 20% of the final course grade.

### Intervention

To gather session-level feedback on the learner experience, we developed a 3-item survey to assess preparation time (9-point scale, from 1 h or less — 5 h or more), familiarity with content from prior courses (5-point scale, not familiar — extremely familiar), and difficulty of working through the materials as measure of cognitive load (5 point-scale, very easy — very difficult) of each prep assignment (Appendix 2). The item on cognitive load has been extensively used in various educational settings; validity evidence has been collected and published to confirm its applicability [30, 31, 36]. All 3 items were assessed by both faculty experts and students to ensure validity in our context. This 3-item PREP survey was included with each RAE starting in 2017. Completing the 3-item survey portion at the end of each RAE was optional and did not contribute to students’ grades. Students were informed that these data were collected for continuous quality improvement during the introduction to the course.

### Measures

The data presented in this study were collected through three consecutive iterations of the course running between August-November in 2017 (Year 1, n = 170), 2018 (Year 2, n = 171), and 2019 (Year 3, n = 171). Students seemed to answer these questions thoughtfully as judged by variation in answers between sessions. Across all three years, only 7 out of 512 students showed little variation in what answers were selected (SD < 0.2), suggesting that they were “straightlining” or providing the same response in each item [31]. These responses were deleted prior to analysis. Two students repeated the course, and their data were deleted from the year they repeated, since they were much more familiar with the content than their peers which is likely to reduce cognitive load and prep time.

### Data analysis

#### Efficiency graphs

**Fig. 1:**
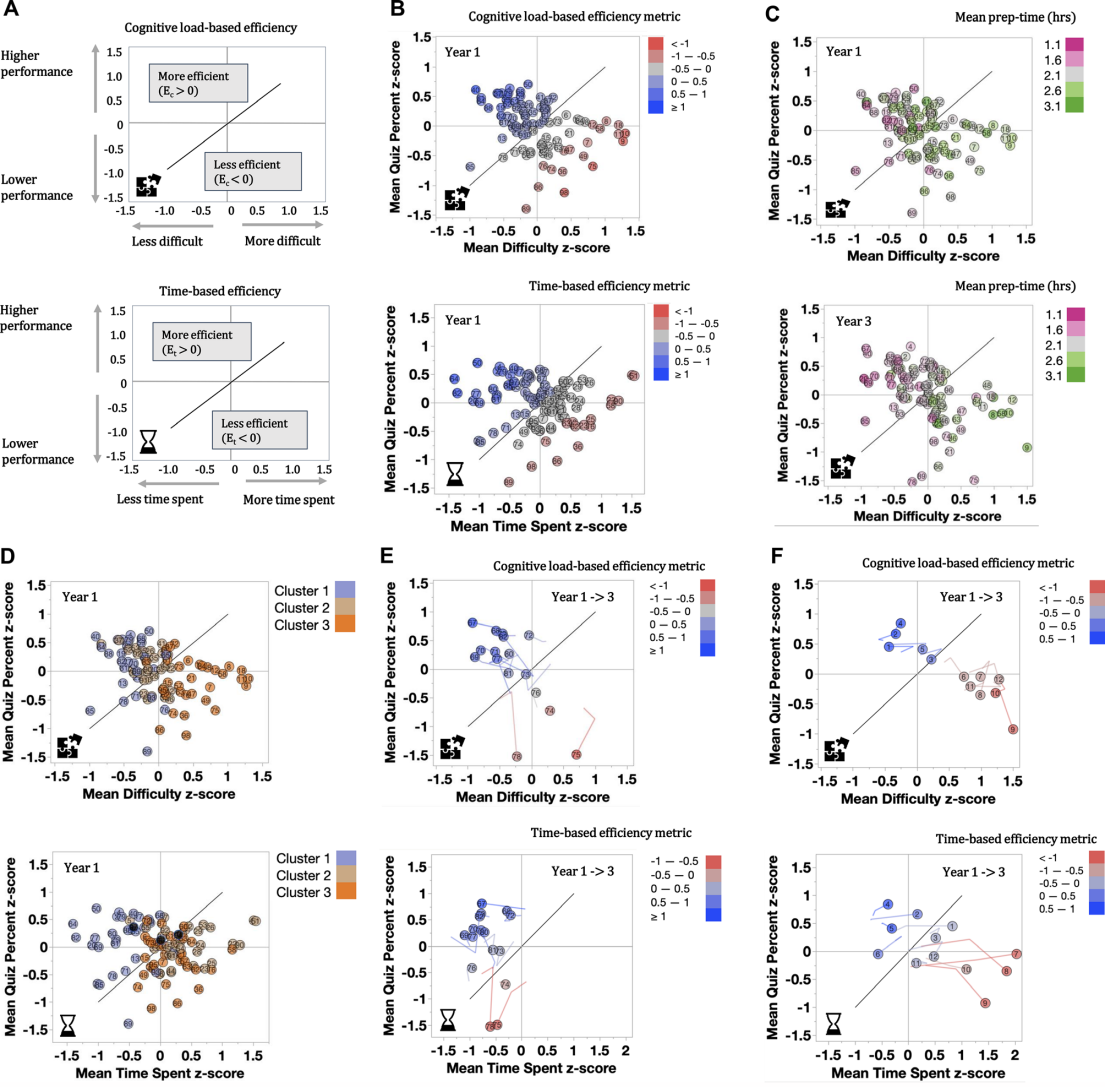
**A) Efficiency graphs (EG).** To produce EGs, the data were standardized by student (z-scores), aggregated by session, and mean session values were plotted. Sessions above the y = x line were considered more efficient, sessions below the line less efficient. The position on the graph with respect to the line can also be expressed as efficiency metric E = (y – x)/√2 [23] **B) Comparing cognitive load- and time-based efficiency in year 1**. Each dot represents one session. Sessions were ordered alphabetically and then numbered from 1–97. To better visualize the position of each session with respect to the line, we colored each dot with the value of the efficiency metric E, for time or cognitive load respectively. In year 1, 25 out of 97 sessions were very efficient (E ≥ 0.5) in either time (n = 7), cognitive load (n = 7), or both (n = 11). Similarly, 27 out 97 sessions were quite inefficient (defined as E ≤ − 0.5) in either time (n = 9), or cognitive load (n = 11), or both (n = 7) **C) Alignment of prep time with cognitive load over the years.** Cognitive load based EGs for year 1 and year 3 were plotted. Each dot represents one session color-coded by prep time in hours. Graphs show a change from year 1 to year 3 in better alignment of prep time with most difficult materials **D) EGs with Cluster overlay.** Cognitive load- and time-based efficiency graphs from panel B were overlayed with the cluster denomination. **E) and F)** **Examples of iterative changes to individual sessions from year 1 to 3 in two different disciplines.** The trail line illustrates the change in position on the graph over the years. The line starts with year 1. The end position in year 3 is indicated by the circular marker.

The item response choices on preparatory assignment time, familiarity and difficulty were converted into numbers (Appendix 2). The data were first standardized by student (z-scores) and then aggregated by session for each year. Expressing the ratings as z-scores reduced variation based on an individual student’s preferences and/or differences in overall ability [27, 30, 31, 36]. The academic performance on the RAE was then plotted versus perceived difficulty to assess the efficiency of the session materials based on cognitive load (Fig. 1A). When plotted in this way, sessions which were most efficient fell in the upper left, above the y = x line where cognitive load was moderate to low, and students performed comparably well on the RAE. Less efficient sessions were in the lower right below the y = x line where cognitive load was higher and/or students performed more poorly.

The position on the plot can also be expressed as efficiency metric *E* – a compound measure (E = y – x)/√2) that describes whether the materials are found above the y = x line (E > 0; more efficient), or below (E < 0; less efficient) [29]. This numeric representation was used to assess sensitivity of the PREP tool (see below). In addition to this traditional cognitive load-based efficiency, we also looked at what we called “time-based efficiency” by exchanging the difficulty rating with time spent (Fig. 1A). By assessing both metrics - cognitive-load and time-based efficiency - educators can determine whether time spent is appropriate for the intrinsic complexity of the topic.

#### Clustering

K means clustering can be used to identify subgroups with common characteristics within a dataset (JMP®, Versions 14–16, SAS Institute Inc., Cary, NC, 1989–2019). We performed K means clustering using raw scores of preparation time, difficulty and familiarity ratings, but excluding quiz performance in order to look at the learning experience independent of outcome. Clusters were generated iteratively for each year and over a range of clusters (numbering from 2 to 5) to determine the best fit based on parallel coordinate plots that can be found in Appendix 3. Unlike with the efficiency graphs raw values were used for the clustering since we wanted to understand the absolute (not relative) values of prep time.

### Instructional design intervention

After the initial end-of-course assessment of the preparatory materials in year 1, the course leadership decided to redesign preparatory materials using an iterative approach. In their end-of-course feedback, students described the student guide, RAEs, and concept videos as very effective, but also described experiencing a lot of variation in the educational quality of individual preparatory materials across sessions and disciplines. Cognitive load theory and multimedia principles provided a framework that allowed us to understand the feedback and how to respond. Table 1 presents a detailed description of how cognitive science and multimedia learning principles informed the iterative improvement of individual preparatory materials over the years. All interventions aimed to optimize intrinsic cognitive load, while reducing extraneous cognitive load [37–39].

**Table 1:** Cognitive load and Multimedia principles used in the redesign of the preparatory materials

Resource	Recommendation for faculty	Applied principle(s)
**Student guide document**	Ensure preparatory questions and keywords are consistent in level of detail, and closely aligned with preparatory resources and in class content. Students were instructed to view the guide first.	Signaling what is important lowers intrinsic cognitive load (Signaling principle^1^, Pre-training principle^2^)
**Online layout**	List preparatory resources in logical sequence with clear directions for students in what order to work through them.	Ordering of learning tasks optimizes intrinsic cognitive load (Simple-to-complex strategy^3^).
**Concept videos**	Provide concept videos where possible. Short (5–10 min), narrated power point presentations were frequently used with emphasis on images and diagrams. Students control playback speed.	Audio/visual information is biologically primary and presents lower cognitive load for novices than written information (Multimedia principle^4^, Segmenting principle^5^, Modality effect^7^).
**Readings**	Revise readings to present concise text with frequent illustrations e.g., instead of assigning a whole book chapter an excerpted chapter was curated.	More focused readings lower extraneous load (Coherence principle^6^), illustrations help optimize intrinsic load (Multimedia principle^4^).
**RAEs**	Add written answers explanations that appear after students have taken the test.	Explanations serve as worked examples, a very effective method to lower intrinsic load for novices (Worked example effect^8^).
**Supplemental information**	Remove any supplemental or optional information from the preparatory resources.	Avoids overlading students with extraneous information (Coherence principle^6^).
**Interactive online modules**	For select topics that require analysis of structures or images consider developing interactive online modules.	Provide additional practice in identifying structures (Variability effect^9^, Transient information effect^10^).
**Dual formatting**	Where possible present content in dual format so student can choose between concept videos and the same material as reading.	Videos lower intrinsic load for novices, students with more expertise may learn better from reading (Expertise reversal effect^11^).
**Self-regulated learning**	Engage students in discussions on instructional design principles and how to use resources most effectively (e.g. role of RAE answers as worked example, dual formatting of readings/videos).	Students need to learn to manage their time and use class resources during prep and consolidation after class (Self-management effect^12^).

**Multimedia Principles**

^**1**^**Signaling principle -** Students learn better when cues that highlight the organization of the essential material are added

^**2**^**Pre-training principle** - Students learn better from a multimedia lesson when students know names/components

^**3**^**Simple-to-complex strategy** - Use learning tasks that first present only isolated elements and gradually increase to full complexity

^**4**^**Multimedia principle** - Students learn better from words and pictures than from words alone

^**5**^**Segmenting principle** - Students learn better from a multimedia lesson that is presented in learner-controlled segments rather than as continuous unit

^**6**^**Coherence principle** - Students learn better when extraneous words, pictures and sounds are excluded rather than included

**Cognitive Load Theory**

^**7**^**Modality effect** - Spoken explanatory text and a visual source of information should be presented at the same time to increase working memory

^**8**^**Worked example effect** - Use worked examples with full solution description that learners can study

^**9**^**Variability effect** - Use learning tasks that differ from one another on all dimension on which tasks differ in real world

^**10**^**Transient information effect** - Self-pacing effect helps students control over the pace of instructional animation

^**11**^**Expertise reversal effect** - Instructional procedures that are designed for novice learners can be counterproductive as expertise increases

^**12**^**Self-management effect** - Teach students to apply CLT principles to manage their own CL to better equip them to deal with preparatory materials

### PREP tool sensitivity

To understand whether the PREP survey was sensitive in identifying changes in the instructional design, we performed a manual audit of the materials for each session independently of the PREP survey results. Only major changes such as adding, removing or replacing resources were considered. In 2018, 28 out of 97 sessions (29%) underwent major revision; in 2019 it was 23 (24%). Of the 51 sessions that were revised, 8 were revised in both consecutive years. We subtracted the differences in *E* scores between consecutive years (Δ), expressed the difference as positive value, and compared the median PREP scores between those materials that had been revised with those that had not been altered using non-parametric analyses. The Mann-Whitney U test and Pearson correlations were performed in JMP. Cohen’s d effect size was calculated [40].

## Results

### Response rate

Students were diligent in completing RAEs in preparing for class. The average response rate for the content-based, graded portion of the RAEs was 98±0.6%. RAEs contained an average of 9±2 content items with a mean item difficulty of 0.88±0.14 (N = 846). Despite the open-book nature of the RAEs, mean item discrimination was 0.36±0.18 (calculated as point biserial, N = 846), indicating students were likely treating the RAE as the low-stakes opportunity to test themselves on their level of preparation that it was meant to be. The average response rate for the optional PREP survey items was 94±4% (time spent), 95±3% (difficulty rating), and 95±3% (familiarity rating). The consistently high response rate suggested that embedding the PREP items into a task that students did routinely minimized survey fatigue.

### Efficiency graphs

We assessed the efficiency of prep materials based on prep time and cognitive load for each session of the course (Fig. 1B). One would expect students to spend more time on content they rated as difficult, but that was not the case. Higher cognitive load efficiency did not necessarily result in lower prep time and vice versa (Fig. 1C), and we found no statistically significant correlation between prep time and difficulty.

### Clusters

To understand better what might determine the allocation of study time, we sought to identify materials that shared common characteristics. Clustering in 3 groups provided the best fit, with statistically significant differences across all parameters (prep time, familiarity, and difficulty) (Table 2).:

**Table 2:** Characteristics of prep materials in each cluster as self-reported by students

Year	Cluster	N of sessions	Prep hours (mean ± SD)	Familiarity (mean ± SD)	Difficulty (mean ± SD)
Year 1	1 (least prep time)	29	**1.8 ± 0.2**	1.7 ± 0.3	2.8 ± 0.2
	2 (most familiar)	31	2.5 ± 0.3	**2.5 ± 0.4**	3.0 ± 0.2
	3 (most difficult)	33	2.3 ± 0.2	1.6 ± 0.2	**3.6 ± 0.3**
		*ANOVA*	*p < .0001*	*p < .0001*	*p < .0001*
Year 2	1 (least prep time)	43	**1.8 ± 0.2**	1.8 ± 0.3	2.9 ± 0.3
	2 (most familiar)	28	2.3 ± 0.3	**2.7 ± 0.3**	3.0 ± 0.2
	3 (most difficult)	25	2.4 ± 0.2	1.8 ± 0.2	**3.6 ± 0.4**
		*ANOVA*	*p < .0001*	*p < .0001*	*p < .0001*
Year 3	1 (least prep time)	39	**1.8 ± 0.2**	1.7 ± 0.3	2.9 ± 0.3
	2 (most familiar)	31	2.2 ± 0.3	**2.5 ± 0.3**	3.1 ± 0.2
	3 (most difficult)	27	2.3 ± 0.4	1.6 ± 0.2	**3.6 ± 0.3**
		*ANOVA*	*p < .0001*	*p < .0001*	*p < .0001*

Ratings from ~ 170 students per year were collected after prep work per session. Session-level data were averaged and clusters were derived by K means clustering (JMP®, Version *14–16*. SAS Institute Inc., Cary, NC, 1989–2021). Familiarity and difficulty were rated on a 5-point scale from least familiar/difficult [1] to most [5]. Analyses of variance (ANOVA) tested differences in means for each individual measure (preparation time, familiarity, difficulty) across clusters within each year. Means and SD shown in bold highlight the value that most distinguished each cluster (least preparation time in cluster 1, most familiar in cluster 2, and most difficult in cluster 3)

- Materials in **Cluster 1** required least prep time, meeting our target of < 2 h on average. They were also perceived as less difficult to learn from even though students were not particularly familiar with the content from courses prior to medical school.
- Materials in **Cluster 2** contained content that students were most familiar with compared to the other two clusters. Materials were rated somewhat more difficult than Cluster 1 materials, but preparation times exceeded our target (average preparation time > 2 h).
- Materials in **Cluster 3** were rated least familiar and most difficult, with average preparation times also exceeding our target (average preparation time > 2 h).

**Fig. 2:**
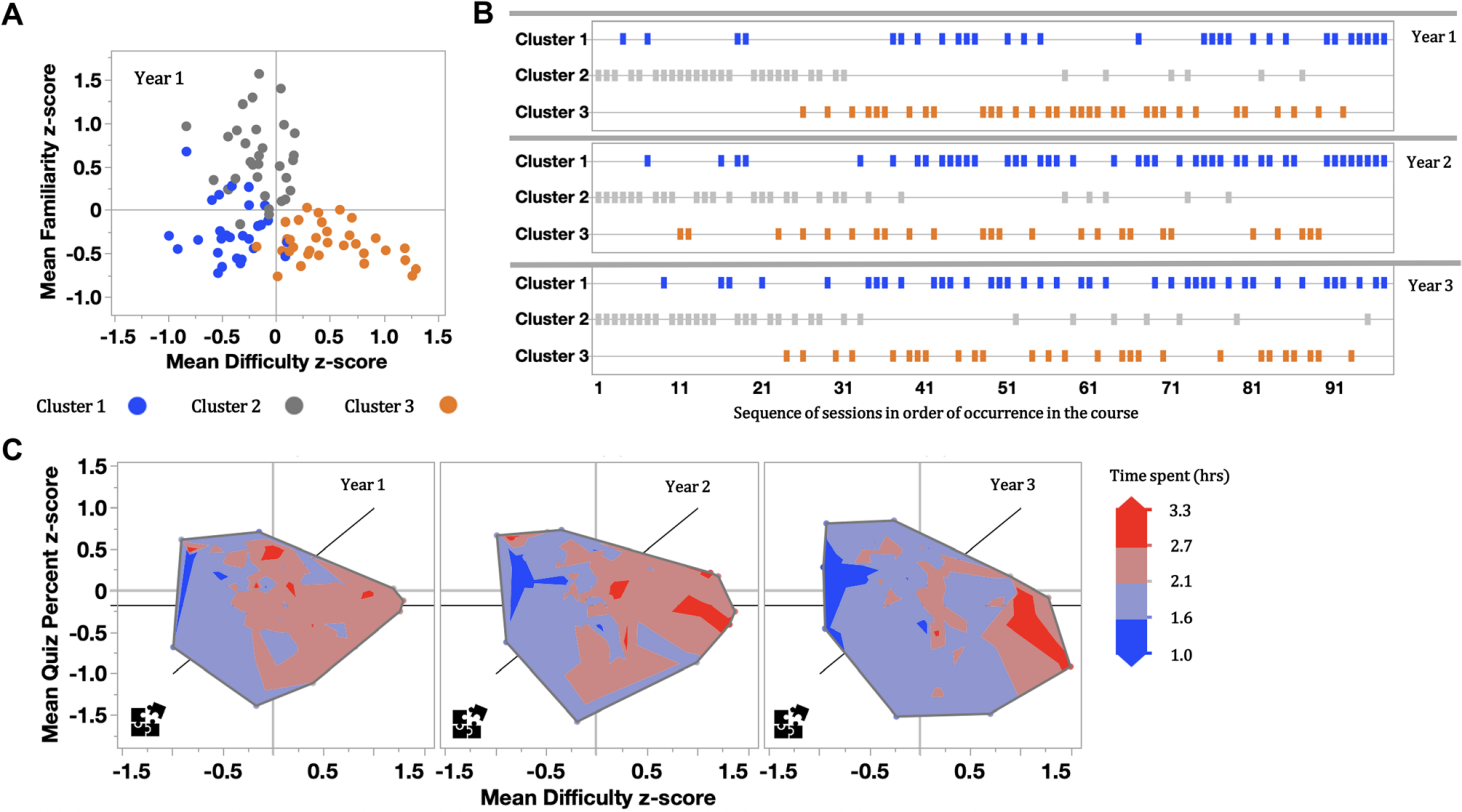
**A) Familiarity.** Familiarity ratings plotted versus difficulty and overlaid with the cluster denomination. The more familiar cluster 2 sessions stood out as a group with moderate difficulty. Cluster 1 and 3 sessions both covered content unfamiliar to the students from prior courses but greatly differed in perceived difficulty of the content **B) Course design**. Sessions were plotted by cluster in the order of occurrence over the time of the course. (Please note that the numbers do NOT correspond to the labels in Fig. 1). Cluster 1 represents content that is unfamiliar and least difficult. Cluster 2 content is most familiar, and moderately difficult. Cluster 3 content is most difficult and least familiar. Preparation times differ across clusters and are discussed in more depth in the text. The course progresses from more familiar to less familiar content over time. Over the years the number of cluster 1 sessions slightly increased (not statistically significant) and cluster 3 sessions were intentionally distributed more evenly across weeks to balance the weekly workload. (A week comprises 8–11 sessions). **C) Impact**. Cognitive load efficiency graphs overlaid with prep time as contour plot highlight how students increasingly invest their time in the most difficult concepts over the years.

Familiar content stood out as a group with moderate difficulty (Fig. 2A). When plotting sessions by Cluster and in sequence of occurrence (Fig. 2B), the more familiar Cluster 2 sessions occurred mostly during first third of the course, while the later part of the course was enriched in Cluster 3 sessions. This indicated a natural progression where earlier parts of the course built more on knowledge acquired prior to medical school than later parts.

While clustering provided us with context for the course, efficiency metrics allowed us to assess which individual sessions to prioritize for improvement. To consider both aspects together, we overlaid the efficiency graphs with our cluster categorization (Fig. 1D). We found that Cluster 1 sessions were both time- and cognitive load-efficient. Cluster 2 sessions were cognitive load-efficient but less time-efficient. Students spent more time on them than one might expect for content that is relatively familiar. Cluster 3 sessions, on the other hand, were least cognitive load-efficient, but counterintuitively more time-efficient. In other words, students spent less time on the materials than seemed appropriate for the most difficult concepts. This suggested that students disengaged from the most challenging materials when making decisions on how to prioritize their study time.

### Demonstrating impact on the course as a whole

Over the next two years, preparatory materials were redesigned iteratively to better align cognitive load- and time-efficiency based on guidelines for faculty described in Table 1. Importantly, in our faculty development efforts we encouraged faculty to apply these strategies as consistently as they could to *all* sessions, while prioritizing Cluster 3 materials for major revisions where possible. In addition, we intentionally spread-out Cluster 3 sessions more evenly to balance the overall workload in each week of the course (Fig. 2B).

Using efficiency metrics, we were able to detect which materials had undergone revision versus those that had not been changed with large effect sizes (Table 3). This suggested that the PREP process was a reliable indicator of changes to the instructional design. Average prep time by session decreased somewhat over three years (from 2.2 to 2.0 h, p < .001), but importantly how students allocated that time changed as well. Sessions that were redesigned based on cognitive load principles (Table 1) showed a better correlation of preparation time with difficulty ratings (r (95) = 0.59, *p* < .0001) compared to those that were not altered (r (91) = 0.32, *p* < .01). The greater alignment of increased preparation time with difficult content is also visualized in Fig. 2C. Average prep time for the more familiar Cluster 2 materials declined (from 2.5 to 2.2 h, p < .003), while prep time for Cluster 1 (1.8 h) and Cluster 3 (2.3–2.4 h) remained the same.

**Table 3:** The PREP survey items detect changes in instructional design with large effect sizes (Cohen’s d)

Difference between years	Prep materials#	Median	Interquartile range	Mann-Whitney U	Cohen’s d
Δ Difficulty z-score	Changes made No change	0.15 0.11	0.08 – 0.27 0.06 – 0.19	*p* < .01	1.1
Δ Familiarity z-score	Changes made No change	0.09 0.07	0.04 – 0.14 0.07 – 0.12		
Δ Time spent z-score	Changes made No change	0.28 0.09	0.11 – 0.55 0.04 – 0.18	*p* < .0001	1.7
Δ Percent score z-score	Changes made No change	0.12 0.08	0.07 – 0.28 0.04 – 0.14	*p* < .001	1.1
Δ Efficiency metric E_c_* (score vs. difficulty)	Changes made No change	0.19 0.10	0.08 – 0.34 0.04 – 0.15	*p* < .0001	1.4
Δ Efficiency metric E_t_* (score vs. time spent)	Changes made No change	0.27 0.08	0.10 – 0.45 0.03 – 0.15	*p* < .0001	2.1
#N = 142 no changes N = 51 changes made 28 sessions were changed in year 2 (out of 97) and 23 in year 3 (out of 96, one session was replaced with a new topic). 97 (Y2-Y1) + 96 (Y3-Y2) = 193 total, 51 changed, 142 no change *E is calculated as (y – x)/√2. If E > 0 materials were considered efficient for learning, if < 0 they were considered less efficient for learning (Paas 2003).					

We reviewed the preparatory materials for each session across all years and marked which ones underwent revision vs. those that did not change. We subtracted the differences in scores between consecutive years (Δ). Differences were expressed as positive values and compared using non-parametric analysis. The tool was found to be capable in detecting differences in both time efficiency and performance efficiency. Familiarity with materials from prior courses did not change based on changes in the instructional design.

In summary, changes in instructional design succeeded in shifting students’ allocation of preparation time away from the more familiar content towards the more difficult content.

### Demonstrating impact on individual sessions

While the PREP process proved useful to assess the course as a whole, it was equally helpful in assessing individual sessions. Figure 1E illustrates the effect of iterative changes in one discipline consisting of 16 individual sessions that were overseen by one content expert. Over the course of two years the reading materials provided to the students as prep resources were shortened. Overall, the materials in this discipline were rated as very time-efficient. But for two sessions (#75 and #78), RAE performance dropped precipitously, suggesting that important information may have been omitted in the process of shortening the content, or that the RAE items were now otherwise misaligned with the revised content. This example demonstrates how efficiency metrics can be used to distinguish intended from unintended consequences, and provide faculty with suggestions for improvement without the need for content expertise.

Figure 1 F demonstrates another set of sessions in a different content area, including some of the most difficult sessions in the course per student ratings. Faculty reviewed the content and confirmed that these sessions covered very complex materials. They were concerned about the apparent lack of engagement with the materials indicated by the comparably low prep time ratings. Over the course of two years, some of the preparatory resources were converted to interactive online modules. These changes successfully increased student engagement with the content as measured in prep time.

Given the overall time constraints of the curriculum, we conclude that the instructional design interventions succeeded at both balancing and somewhat reducing overall workload while redirecting available time towards the more difficult concepts, in other words – it is prep time well spent.

## Discussion

The efficiency metrics used in PREP allow educators to identify preparatory resources based on their learner’s cognitive load and available time. We present this study as proof of concept that the PREP can be used to assess and improve preparatory materials in the flipped classroom and as such presents a novel tool for course evaluation that is based on educational theory [31].

PREP was sensitive in detecting changes to the instructional design without the need for content expertise. This made it a particularly useful tool in the context of our multidisciplinary settings. The familiarity measure proofed helpful in guiding sequencing and integration of course materials from more familiar to less familiar from prior courses, an important course design principle to optimize intrinsic load [26]. The metrics of time- and cognitive-load efficiency proved meaningful in identifying specific resources in need of revision. By expanding the efficiency concept to include self-reported prep time, a behavioral outcome measure of engagement with the materials, we expanded the utility of this approach to help address the long-standing problem of balancing content- and time-constraints in preclinical medical education [5, 7].

Based on iterative revisions grounded in CLT (Table 1), we succeeded in engaging our students on spending less time on more familiar content and focusing their time on materials that were conceptually more difficult. This is consistent with the literature. The learning process is prone to many cognitive biases and illusions, such as fluency in recalling factual information, that can mislead students to think that learning has been achieved and also can interfere with learning [26, 41]. The success of the flipped classroom approach depends on learners preparing independently and therefore raises the stakes for instructional design. A recent review highlights the need for clearly structured, interactive, and engaging out-of-class assignments for the flipped classroom to succeed [42, 43]. PREP provides educators with a framework and a tool to identify preparatory assignments that need revision and track the impact of these changes in the quality of the out-of-class assignments.

The need of novice learners for structure and scaffolding [5, 26] is easily misunderstood by educators as not wanting to put in the effort to learn. The goal of this work is not to create shortcuts or “cheat-sheets” for learners. Cognitive load theory explicitly states that the intrinsic cognitive load of a topic cannot be changed [26]. The goal is the opposite, to sustain the learner’s attention such that they stick with the hard topics. The science of instructional design helps us to support our learners to better manage their learning and make it easier to prioritize difficult content [26]. After almost a decade of experience with flipping the entire pre-clinical curriculum [44], our experience suggests that if we are committed to active learning, we must also be committed to effective instructional design of the preparatory assignments. Although developed and studied within a specific curriculum, we believe this method is relevant to other flipped-classroom settings.

### Limitations

This study presents a quality improvement project conducted at a single intuition and as such the specific data are not generalizable. For example, our finding of 2-hour prep time being time-efficient, might be 1 or 3 h in a different curricular context. However, we believe that the PREP process itself is likely of general interest to educators in medical and higher education. Unlike traditional end-of-course evaluations, PREP data are collected in near real-time and grounded in educational theory. As such the PREP process provides highly detailed and actionable insights into the “cognitive landscape” of the course from the perspective of the learner. The strength of this approach is its high ecological validity, though the ratings provided by the students might be prone to various biases. While we have observed a reasonable degree of variation in the data and took care to normalize by student to mitigate effects based on prior educational experience, we cannot be certain how much thought each student gives the ratings at each time. The approach may also not be useful for small classes. Future studies should look at how learners with different backgrounds, ethnicity or socioeconomic status might differ in their experience of the course.

Despite many changes made to individual study resources, the learning objectives taught throughout the three years of session-level data collection were the same. The effect of changes made to individual sessions varied, some having the intended outcomes, others indicating further need for improvement. Furthermore, the efficiency graph approach assumes that the RAE effectively measures the knowledge students acquire during prep. Select items in 9 RAEs (5 in year 2, and 4 in year 3) underwent significant revision along with changes made in prep resources. We think it unlikely that the changes to the course overall are an artifact of these specific edits to select RAE items, but for conclusions on individual sessions it will be important to take alterations in RAE content into account.

## Conclusion

The iterative cycle of curriculum or course improvement is routinely performed by faculty and requires significant time and resources. Yet, this work is often performed based on subjective impressions and typically lacks outcome data grounded in educational theory. The success of the flipped classroom approach depends on learners preparing independently and therefore raises the stakes for instructional design. Our data-driven PREP approach provides educators with an analytic process focused on the two most challenging domains for novice learners – cognitive load and managing time. Efficiency metrics allow educators to improve instructional resources based on their learner’s cognitive needs and available time. In addition, they provide an opportunity for educators to manage and prioritize their own time in revising content, as well as to demonstrate the impact of continuous curricular quality improvement to students, colleagues and administrators in ways that are otherwise intractable. We believe that session-level approaches like PREP fill an important gap in assessing curricula not captured in traditional satisfaction-based course evaluations.

## Electronic supplementary material

Below is the link to the electronic supplementary material.

Supplementary Material 1

Supplementary Material 2

Supplementary Material 3

Supplementary Material 4

## Data Availability

The data that support the findings of this study are available from the corresponding author upon request.

## Declarations

### Prior presentation

Parts of this manuscript were presented at the Harvard Initiative for Learning and Teaching innovation showcase in April 2019, at the International Association of Medical Science Education annual meeting in June 2021, and at the AMEE Lyon 2022 annual meeting in August 2022.
