# Supplementary material for: Using cognitive load theory to evaluate and improve preparatory materials and study time for the flipped classroom
Source: BMC Med Educ. 2023 May 17;23:345. doi: 10.1186/s12909-023-04325-x (PMC10193725; doi:10.1186/s12909-023-04325-x)

**Appendix 1. A typical week in the Foundations course.** The course interleaves multiple content areas. There are three in-class sessions on Monday, Tuesday, Thursday, and Friday. Students are encouraged to prepare for two in-class sessions per day, and complete review and self-assessment each day as part of their preparatory work. Dark colors represent in-class sessions, light colors represent preparation for in-class sessions. POM = Practice of Medicine Course.


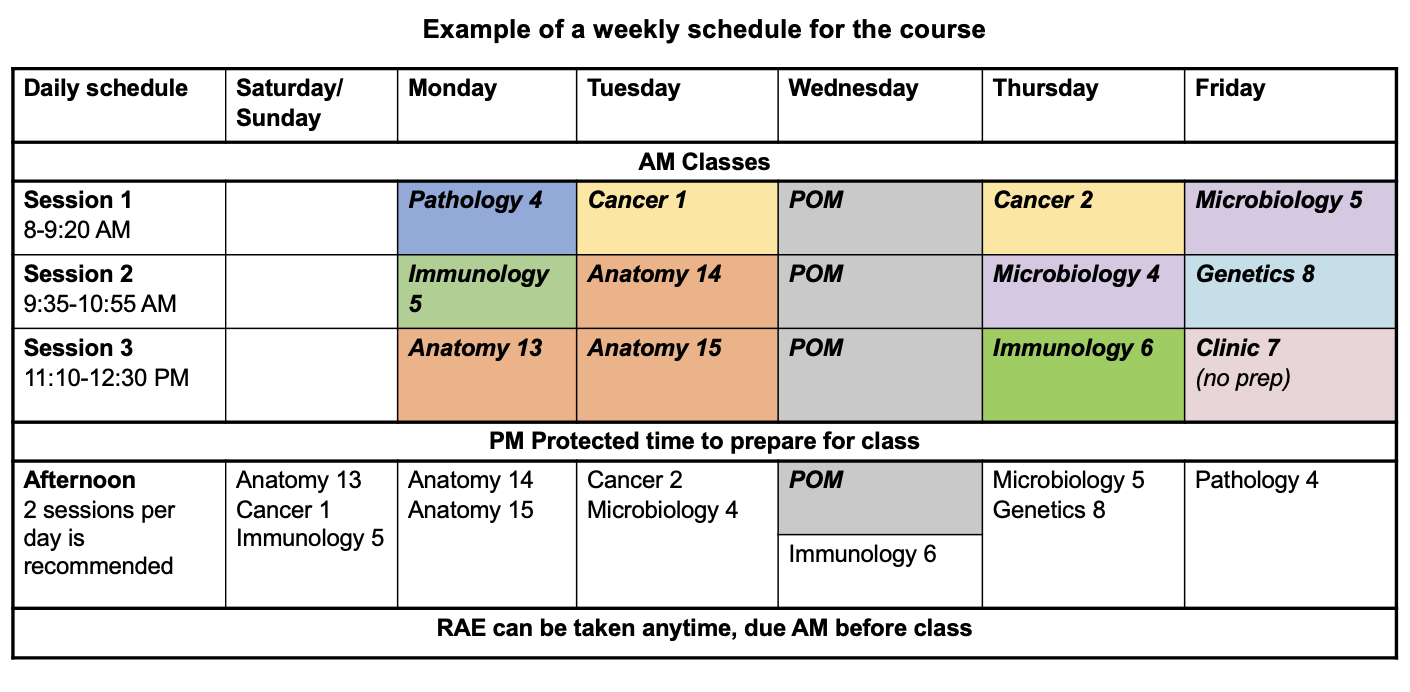

Supplement: Supplementary file 1 — Supplementary Material 1 [file 12909_2023_4325_MOESM1_ESM.docx]
