# Supplementary material for: Using cognitive load theory to evaluate and improve preparatory materials and study time for the flipped classroom
Source: BMC Med Educ. 2023 May 17;23:345. doi: 10.1186/s12909-023-04325-x (PMC10193725; doi:10.1186/s12909-023-04325-x)
Supplement: Supplementary file 3 — Supplementary Material 3 [file 12909_2023_4325_MOESM3_ESM.docx]

**Appendix 2 – RAE survey items**

Data were collected using ordinal scales shown below, and later re-coded as values indicated in brackets.

How long did it take you to prepare for this session?

- 1 hour or less (1)
- 1.5 (2)
- 2 (3)
- 2.5 (4)
- 3 (5)
- 3.5 (6)
- 4 (7)
- 4.5 (8)
- 5 hours or more (9)

How difficult was it to learn from the prep materials?

- Very difficult (5)
- Difficult (4)
- Neutral (3)
- Easy (2)
- Very easy (1)

How familiar were you with this content from previous course work?

- Not at all familiar (1)
- Slightly familiar (2)
- Somewhat familiar (3)
- Moderately familiar (4)
- Extremely familiar (5)
