# Supplementary material for: Using cognitive load theory to evaluate and improve preparatory materials and study time for the flipped classroom
Source: BMC Med Educ. 2023 May 17;23:345. doi: 10.1186/s12909-023-04325-x (PMC10193725; doi:10.1186/s12909-023-04325-x)

**Appendix 3. Cluster details and parallel coordinate plots**

Cluster details (mean and stdev) are also presented in Table 3. The parallel coordination plots provide a graphic depiction of the individual data points in each cluster. The fourth plot in the lower right-hand corner depicts the cluster means for each value for visual comparison. Please note that these plots are generated automatically by JMP^®^ (Versions 14-16, SAS Institute Inc., Cary, NC, 1989-2019), and the colors do not correspond to those in Figure 2. The parallel coordinate plots show that materials in red (cluster 2) were rated most familiar and less difficult but prep times were just as high as with the least familiar content in blue (cluster 3). Then there was a third group (green/cluster 1) that despite being unfamiliar was rated as least difficult and lowest in prep time.

Red/1 = cluster 2 (grey in Figure 2)

Green/2 = cluster 1 (blue in Figure 2),

Blue/3 = cluster 3 (orange in Figure 2).


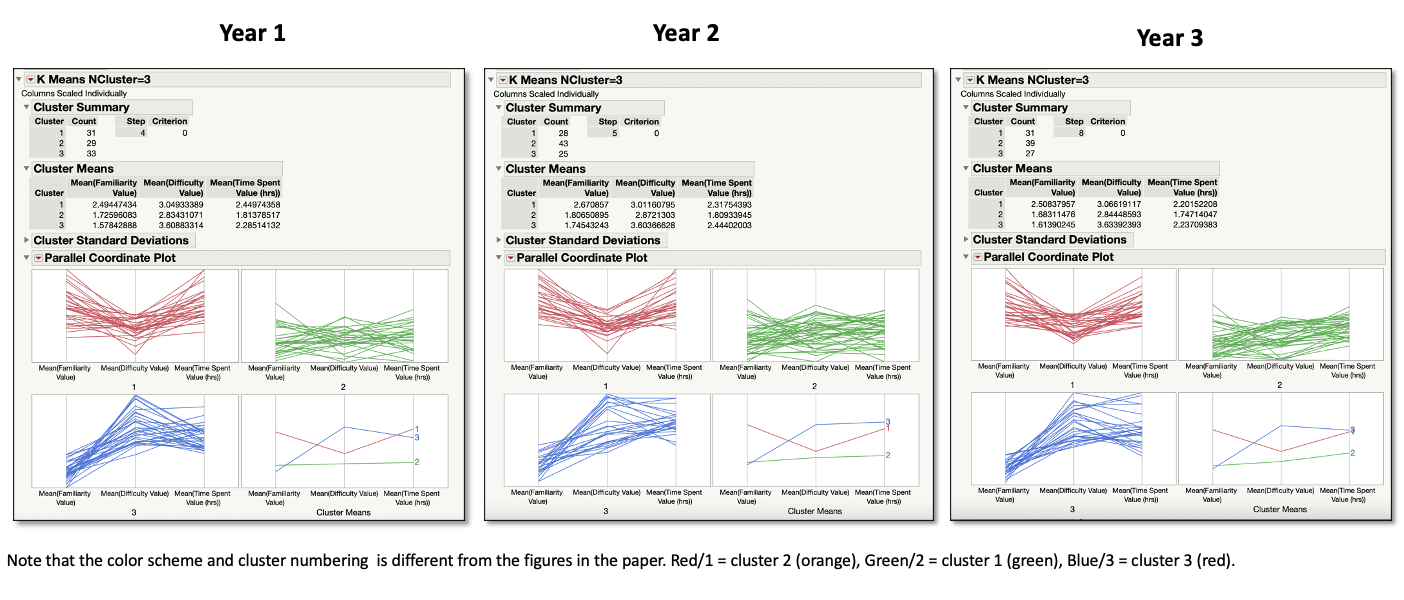

Supplement: Supplementary file 4 — Supplementary Material 4 [file 12909_2023_4325_MOESM4_ESM.docx]
